# MALDI-TOF mass spectrometry on intact bacteria combined with a refined analysis framework allows accurate classification of MSSA and MRSA

**DOI:** 10.1371/journal.pone.0218951

**Published:** 2019-06-27

**Authors:** Wenhao Tang, Nisha Ranganathan, Vahid Shahrezaei, Gerald Larrouy-Maumus

**Affiliations:** 1 Department of Mathematics, Imperial College London, London, United Kingdom; 2 MRC Centre for Molecular Bacteriology and Infection, Department of Medicine, Faculty of Medicine, Imperial College London, London, United Kingdom; 3 MRC Centre for Molecular Bacteriology and Infection, Department of Life Sciences, Faculty of Natural Sciences, Imperial College London, London, United Kingdom; H Lee Moffitt Cancer Center and Research Institute, UNITED STATES

## Abstract

Fast and reliable detection coupled with accurate data-processing and analysis of antibiotic-resistant bacteria is essential in clinical settings. In this study, we use MALDI-TOF on intact cells combined with a refined analysis framework to demonstrate discrimination between methicillin-susceptible (MSSA) and methicillin-resistant (MRSA) *Staphylococcus aureus*. By combining supervised and unsupervised machine learning methods, we firstly show that the mass spectroscopy data contains strong signal for the clustering of MSSA and MRSA. Then we concentrate on applying supervised learning to extract and verify the important features. A new workflow is proposed that allows for extracting a fixed set of reference peaks so that any new data can be aligned to it and hence consistent feature matrices can be obtained. Also note that by doing so we are able to examine the robustness of the important features that have been found. We also show that appropriate size of the benchmark data, appropriate alignment of the testing data and use of an optimal set of features via feature selection results in prediction accuracy over 90%. In summary, as proof-of-principle, our integrated experimental and bioinformatics study suggests a novel intact cell MALDI-TOF to be of great promise for fast and reliable detection of MRSA strains.

## Introduction

As more antibiotic-resistant pathogens emerge, fast, accurate, robust and reliable detection of bacterial pathogens as well as their resistance to antibiotics is urgently needed to contain the spread of these so-called “superbugs”. This is the case for *Staphylococcus aureus*, which is the most common and important pathogen in both community-associated and clinical-associated infectionKock, Schaumburg [1]. Currently, identification and susceptibility testing of *S*. *aureus* isolates is time-consuming (up to 48 hours) and laborious even if PCR-based methods are used to increase the speed of identification and susceptibility[2, 3]. However, in the last 15 years, matrix-assisted laser desorption ionization time-of-flight mass spectrometrya (MALDI-TOF) has become the workhorse of most clinical microbiology laboratories[4]. The introduction of MALDI-TOF, based on protein profiling, has completely revolutionized the identification of pathogens. However, prior to analysis by MALDI-TOF, sample preparation can require several steps and identification relies mainly on identification of intracellular proteins[5, 6]. In this study, we propose a new route to identify methicillin-susceptible (MSSA) and methicillin-resistant (MRSA) *Staphylococcus aureus* infection, based on direct peptide fingerprints of intact heat-inactivated *S*. *aureus* clinical isolates using an improved data analysis workflow and machine learning.

We introduced an improved workflow to analyse our mass spectroscopy (MS) datasets. In particular, our new workflow extracts a fixed set of reference peaks from the training data. Then given any new test data, they can be pre-processed independently and mapped onto the reference peaks. Furthermore, we examined two different ways to generate a feature matrix (intensity-based and peak area-based methods) and two different statistical learning models (random forest[7] and binary discriminant analysis (BinDA[8])). In addition, we created training and testing datasets in two different ways such that the training procedure was conducted with or without the biological replicates of the testing data (technical replicates of testing data were never included in the training procedure). The high accuracy rates observed suggests that the consistent feature matrix generated by our proposed workflow provides efficient processing of data for classification. Finally, we selected top ranked peak positions and discuss their possible biological origin. Overall, our combined experimental and bioinformatics approach could potentially be used for fast and reliable clinical diagnosis of infection with antibiotic resistant strains of *S*. *aureus*.

## Materials and methods

### Bacterial strains and conditions

For the present proof-of-principle study, we examined 10 methicillin-susceptible *S*. *aureus* (MSSA) and 10 methicillin-resistant *S*. *aureus* (MRSA) clinical isolates recovered from individual patients with bacteraemia by the diagnostic microbiology service at Charing Cross Hospital. The strains were grown in Tryptic Soy Broth (TSB) medium at 37°C for 12 hours to reach an OD_600_ of 0.4–0.6.

### Sample preparation

Bacteria were harvested, heat-inactivated 30 mins in at 80°C, washed three times and suspended in double distilled water at a final concentration of 10^4^ bacteria per μl.

### Experimental design

As proof of principle, we generated a total of 7 datasets corresponding to four biological replicates and three of these contained two technical replicates (see Table 1). Each dataset contained 20 clinical samples: 10 MRSA and 10 MSSA samples. To be more specific, the 1–10 MRSA/MSSA samples in Data 1 are technical replicates of 1–10 MRSA/MSSA samples in Data 2 respectively; The 1–10 MRSA/MSSA samples in Data 1 and another 1–10 MRSA/MSSA samples in Data 3, 4, 5, 6 and 7 are biological replicates respectively.

**Table 1:** Experimental data used throughout the paper. Each dataset consists of 10 MRSA and 10 MSSA samples.

	Biological replicate 1	Biological replicate 2	Biological replicate 3	Biological replicate 4
Technical replicate 1	Data 1	Data 3	Data 5	Data 7
Technical replicate 2	Data 2	Data 4	Data 6	

### MALDI-TOF analysis

Prior to mass spectrometry analysis, the 2,5-dihydroxybenzoic acid (DHB) matrix was added to a final concentration of 10 mg/mL in chloroform/methanol at a ratio of 90:10 v/v. 0.4 μL of cell solution and 0.8 μL of the matrix solution were deposited on the MALDI Target, mixed with a micropipette and left to dry. MALDI-TOF analysis was performed on a 4800 Proteomics Analyzer (with TOF-TOF Optics, Applied Biosystems) using the reflectron mode. Samples were analyzed operating at 20 kV in negative and positive ion mode, and three independent experiments were performed. Mass spectrometry data were analyzed using both Data Explorer version 4.9 from Applied Biosystems and R [9].

**Fig 1:**
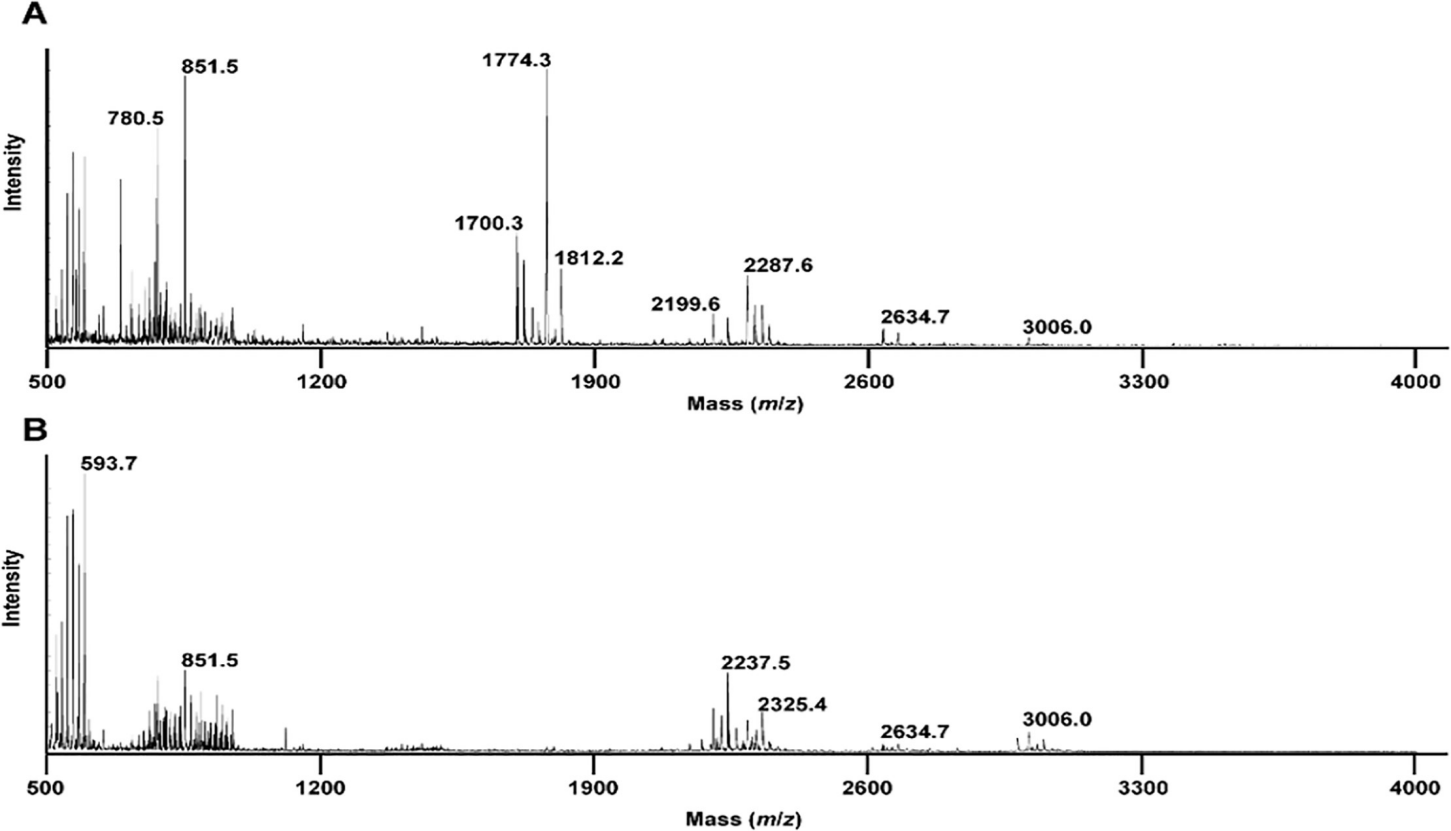
MALDI-TOF positive ion mass spectra of MSSA (A) and MRSA (B) in the reflectron mode. Bacteria were grown on TSB medium, washed three times with double-distilled water and loaded into the MALDI target followed by the addition of the matrix directly on the bacteria.

The MS data for each sample consists of around 130,000 pairs of (*m/z*, *I*) values, where *m/z* stands for the mass to charge ratio and *I* stands for the intensity value. *m/z* values range from around 399 to 4012 (Fig 1). Since there is variation in *m/z* values across samples and experiments, we cannot directly summarize the MS data into a *M*×*N* feature matrix of *M* features (peak positions or *m/z* values) and *N* samples and several steps of pre-processing is required as explained in “Data pre-processing”.

## Results

### Identification of peptide markers for discrimination between MSSA and MRSA strains

Several methods have been reported to identify bacteria based on their protein fingerprint[4, 10], here we opt for a direct identification of intact heat-inactivated bacteria using 2,5-DHB as the matrix solubilised in organic solvent and MALDI-TOF MS in the positive reflectron mode. Using this approach, the most intense peaks appeared in the range *m/z* 500 to 3000. In the case of MSSA (Fig 1A), the representative mass spectrum is dominated by 5 sets of peaks in the range *m/z* 500–900, *m/z* 1650–1850, *m/z* 2100–2300, *m/z* 2600–2700 and *m/z* 2900–3100. The peaks in the ranges *m/z* 500–900, *m/z* 2600–2700 and *m/z* 2900–3100 remain to be assigned. However, according to the literature and MS/MS data (not shown), the peaks found at *m/z* 1650–1850 can be assigned to the peptide fragment identified as the phenol-soluble modulin α1Δ1–5 (PSMα1Δ1–5) with the sequence GIIKVIKSLIEQFTGK with a theoretical *m/z* of 1774.083 [11]. The peaks found in the range *m/z* 2100–2300 are assigned to formylated peptides such as PSMα1 at *m/z* 2287.3 (theoretical *m/z* 2787.346). In the case of MRSA (Fig 1B), the representative mass spectrum is dominated by 4 sets of peaks in the ranges *m/z* 500–900, *m/z* 2100–2300, *m/z* 2600–2700 and *m/z* 2900–3100. Interestingly, compared to MSSA (Fig 1A), in MRSA, (Fig 1B), the intensity of the set of peaks ranging from *m*/*z* 1650 to m/z 1850 decreased drastically. It is worth to mention that in the experimental setting used by Lu and colleagues, which consisted in performing an extraction of surface proteins and peptides by using 70% ethanol followed by addition of 70% formic acid and acetonitrile to the supernatant obtained, such peptide markers have also been observed in the extract of the reference strains MSSA ATCC2913 and MRSA N315 [11]. Thus, giving confidence in the specificity of the ones found using our methodology.

### Data pre-processing

Various methods have been proposed for pre-processing and extraction of features from MS data. Yasui et al.[12] proposed a workflow for pre-processing Surface Enhanced Laser Desorption/Ionization (SELDI) data. They tried to summarize MS data into local peak/non-peak binary data instead of relying on the absolute intensity values as biomarkers. Tibshirani et al.[13] log-transformed the intensities and removed baseline at first, they then normalized each spectrum by a linear transformation which mapped the 10th and 90th percentiles to 0 and 1. After this, they detected peaks in a similar way to Yasui et al.[12] and aligned peaks by clustering, which results in common peaks in individual spectra. Based on the common peaks, they found the most discriminatory threshold which helps generate a binary feature matrix in the end. However, their peak detection method is crude since the isotopic envelop issue may not be resolved clearly[14]. Gibb et al.[15] proposed a comprehensive workflow for pre-processing MS data and also developed an R package called “MALDIquant” for implementing MS data analysis.

Binning the peak positions or their intensities is a common step in MS data pre-processing[12, 15]. The basic idea of binning is to either summarize peak positions or the corresponding intensity values within a certain range of a certain peak position into a scalar. The details of binning implementations can vary. Although binning has been widely applied, it has also been criticized in some papers. Key et al.[16] argued that binning is not a good idea due to the existence of outliers. Hansen et al.[17] argued that the methods chosen to decide the number of bins or the bin size and how to place bins remain a problem. Even though binning intensities seems to still be a necessary step in MS data analysis, it can act as a very simple dimensional reduction method as long as all the spectrums are well aligned. In this paper, we adapt “MALDIquant” to align spectra. The alignment method used in MALDIquant has been described previously by He et al.[18]

**Fig 2:**
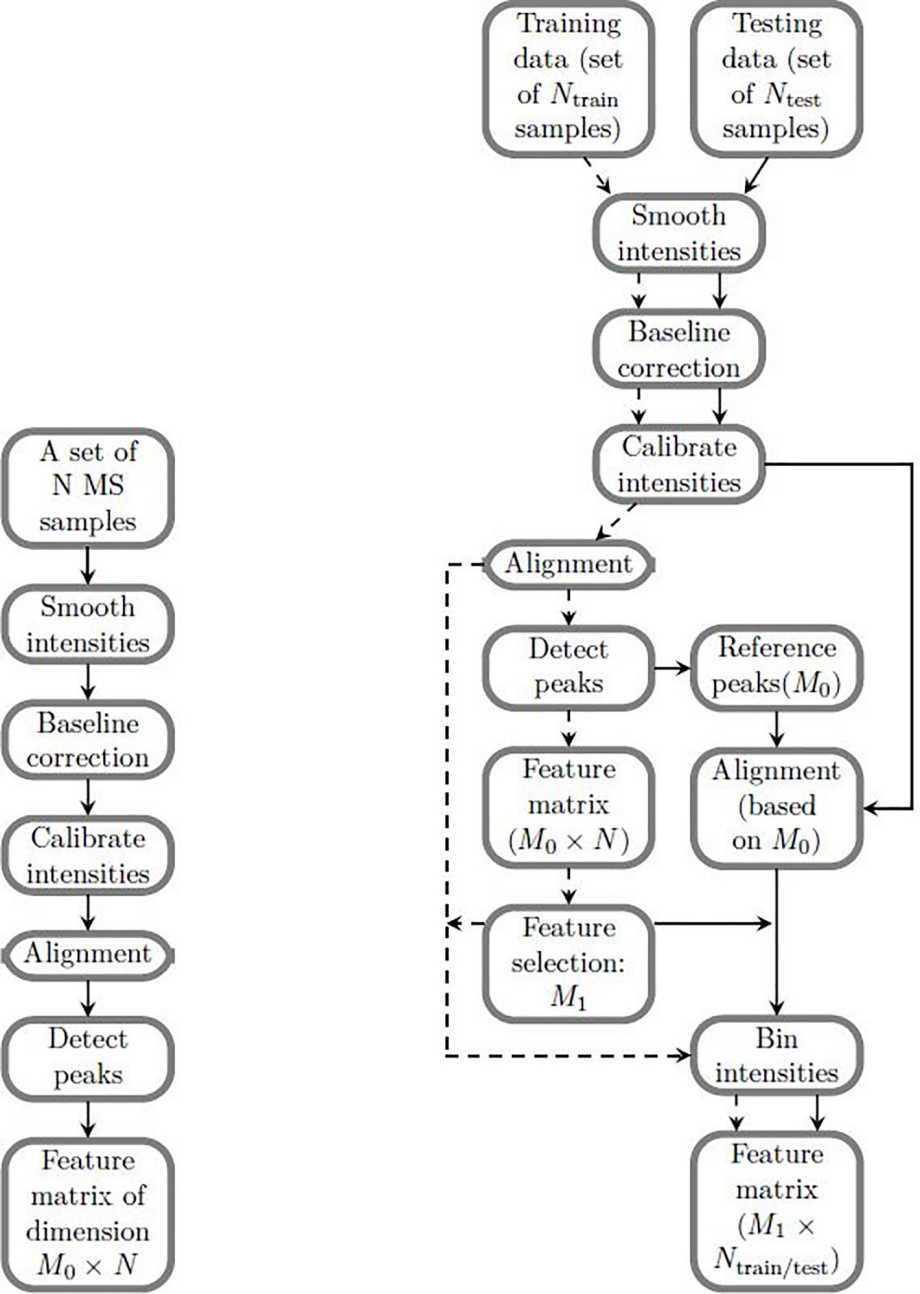
Two workflows used in this paper. (A) Workflow suggested by Gibb et al.[15] (B) Workflow that allows selecting important features and building a statistical model for supervised learning. The feature matrix generated from testing data is consistent with the feature matrix generated from training data when using this workflow.

An issue with current MS data pre-processing workflows is that we cannot generate consistent feature matrices for new datasets. In practice, we would like to develop the statistical model based on some benchmarking dataset and then apply it to data from new samples. If we followed the first workflow on two MS replicates independently, the two feature matrices would consist of different numbers of features. Even if the numbers of features are the same, the features are not exactly the same. This is because different peak lists are detected and hence different set of reference peaks are generated. We would like to summarize different MS replicates into a consistent feature matrix so that we can further apply supervised learning methods and check the robustness of the features that we used. Such difficulty can be resolved by using a reference-based workflow. In this paper, we apply two different workflows for pre-processing and analysis of the MS data (Fig 2). We evaluate the first workflow proposed by Gibb et al[15] (Fig 2A) using unsupervised learning and a combination of both supervised and unsupervised method to reveal that there is strong signal which allows for classification of two kinds of bacteria. We then proceed to extracting and evaluating the reference features using the second workflow (Fig 2B). The detailed parameter settings of two workflows will be discussed at the end of this subsection. For the second workflow, we assume that the testing data is not available in the identification of the feature matrix and reference peaks from the training data. Based on the training data, we generate a vector of peak positions as a reference: $R=\{R_{1},R_{2},\ldots ,R_{M_{0}}\}$. Details about how to extract the reference peaks will be discussed later in this subsection. Based on the reference *R*, we aligned the testing MS data and summarized aligned MS data into a feature matrix. We have two choices for summarizing aligned MS data: using either intensity based or peak area based feature matrices:

- For the intensity based feature matrix we used the function “match.closest” from R package “MALDIquant”[15] to match the reference peak position to each sample’s peak position. We should note here that samples need to be aligned based on the reference peak position in advance (the impact of such alignment will be discussed in the second part of evaluations of supervised learning study). This step can help alleviate the effect of the strong variation of *m/z* values across different experiments and thus generate a meaningful and consistent feature matrix for both training and testing MS data. Suppose that after alignment, one sample contains the vector of peak positions *X* = {*x*_1_,*x*_2_,…,*x*_*l*_},*l*>*M*_0_. If each *R*_*i*_ was mapped to a unique *x*, then the corresponding *M*_0_ intensities of that sample were used to form the feature matrix. Otherwise, if multiple *R*_*i*_ were matched to the same *x*_*j*_, we simply allocate the intensity of *x*_*j*_ to the smallest mapped *R*_*i*_. For the other unmapped *R*_*i*_ we used interpolated intensity values approximated based on the sample itself.
- For the peak area based feature matrix we set the size of each bin to be 1 *m/z* value: the sum of the intensity values with corresponding *x* that lie within *R*_*i*_±0.5 for each *i*∈{1,2,…,*M*_0_} in each sample. Thus, for each sample we have a vector of length *M*_0_, which forms the feature matrix. Yasui et al.[12] set bin sizes to be ±0.2% around detected peak positions, which means that the bin size increases as peak position increases. In practice, we found that this method also led to good results, but 0.2% is too large an error, resulting in a big overlap between neighbouring bins at high intensity values.

The detailed parameter settings and algorithm used in the R package MALDIquant are as follow: For the preprocessing of training data, the raw data were smoothed using moving average method and half window size set to 2. The baseline was removed using method “TopHat” with half window size set to 10. The intensities were calibrated using “TIC” (Total Ion Count) method. Then the data were aligned with half window size set to 20, tolerance set to 0.02, “minFrequency” set to 0.75 and signal-to-noise-ratio (SNR) set to 10.

After the above procedure, we were able to extract reference peaks. Firstly, we detected peaks based on the aligned training data by setting SNR to 2 and half window size to 20 (using median absolute deviation (MAD) method). Then the reference peaks were created by calling the function “referencePeaks” from MALDIquant with method “strict”, “minFrequency” set to 0.75 and tolerance 0.02. To be more specific, the mass of peaks detected from each sample were gathered as a vector and ordered. Then the differences between each neighbor were calculated. After that, starting from the largest gap, the vector of masses was repeatedly divided into bins until the bin does not contain two or more peaks of the same sample and all peaks in a bin are near to a specific position which was determined by the parameter tolerance [15].

For the preprocessing of testing data, it was also smoothed, baseline corrected, intensities and calibrated using the same parameter setting as illustrated above. In alignment, the reference peaks extracted from the training data were specified.

After preprocessing the data, we were able to generate either peak area or intensity based feature matrix.

### Supervised learning

Given a feature matrix and corresponding true labels, there are also different ways of performing feature selection. Support Vector Machine with Recursive Feature Elimination (SVM-RFE) was proposed by Guyon et al [19]. Tim et al. [20] have proposed a feature selection method using compressive sensing, which aims to find a classifier that is as sparse as possible. Gibb et al. [8] have proposed BinDA, which is a generalization of “PPC” [13]. Random forest can be also used for ranking features. In this paper, we focus on BinDA (BI) and random forest (RF) for selecting top *M*_1_ ranked features from the *M*_0_ full features and building a statistical model for testing (*M*_1_≤*M*_0_).

Each one of three evaluation procedures was performed using the following 4 different variants of the second workflow:

1. Generating an intensity-based feature matrix and transforming it to a binary matrix for supervised learning using BinDA (“I+BI” for short).
2. Generating an intensity-based feature matrix and building a random forest model for testing (“I+RF” for short).
3. Generating a peak area-based feature matrix and transforming it to a binary matrix for supervised learning using BinDA (“P+BI” for short).
4. Generating a peak area-based feature matrix and building a random forest model for testing (“P+RF” for short).

Apart from the above four variants, all the parameter settings used in MALDIquant for intensity smoothing, baseline correction, intensity calibration and alignment are kept the same.

We also tried to incorporate the first workflow into supervised learning framework using the same parameter settings as illustrated above. Procedures with BinDA or random forest are termed as “MALDI_BI” and “MALDI_RF” respectively. We combine all training samples and one testing sample (leave one out) and obtain the feature matrix using MALDIquant. Then a subset of feature matrix was used for training and selecting the top ranked features for testing the one testing sample. This procedure was repeated for each one of samples used for testing. The “leave one out” procedure could potentially reduce the impact of testing sample on the alignment step. We should note that the limitation of using the first workflow in supervised learning is that the features could change due to different input data. Because different peaks can be detected and hence reference peaks are not the same. The procedure with.

For supervised learning, there are two ways of creating training and testing data: by splitting datasets or samples, which correspond to training with or without biological replicates of testing data in training procedure (technical replicates of testing data are always excluded in training procedure).

The evaluation of the workflows in terms of supervised learning focuses on three parts. The first part is deciding upon the top ranked features used in supervised learning. There are 8 different choices for selecting 4 out of 7 datasets such that the 4 datasets are all biological replicates. In order to create training and testing data, if we split datasets, for each of the 8 subsets, we conducted supervised learning based on the 3 datasets ((43)×8=32 choices in total). If we split samples, in each dataset, we randomly selected 7 MRSA and 7 MSSA samples for constructing training dataset, so that 56 out of 80 samples were selected to form training data, this bootstrapping was repeated 10 times under each one of 8 subsets. When splitting samples, we made sure that biological replicates of the samples included in testing procedure do not appear in training datasets. Only the top K (*K*∈{5,10,15,30,50,100,300,500,1000,Full features}) ranked features were kept for training and testing.

The second part concerns the supervised learning results using the top 100 ranked features. As above, we can generate training and testing data by either splitting datasets or samples. For the former case (splitting datasets), each one of 7 datasets were set as testing data. We gradually increase the size of subset of the remaining datasets for training and make sure that technical replicate of the testing data does not appear in the training data. For the latter case (splitting samples), for each one of 8 subsets as mentioned before, we split samples into training and testing data by the following procedure: In each biological replicate, 3 MRSA and MSSA samples were randomly selected for constructing testing dataset (hence testing data contains (3+3)×4 = 24 samples). As above, during splitting we made sure that technical replicates of testing samples were also excluded from training procedure. This way of constructing testing data results in 56 samples left. Among the 56 samples, we randomly selected subset of samples with size 8, 16, 24, 32, 48 and 56 for training. In addition, we made sure that in both training and testing data, MRSA/MSSA samples were evenly spread. This splitting was repeated under each one of 8 subsets 5 times, which results in 40 accuracy rates for each one of 4 different variants of the second workflows as discussed below.

The third part concerns the evaluation of the stability of the top ranked features. We expect a consistent pattern of extracted features based on different training datasets. The top 100 ranked features were extracted based on training datasets which were obtained by splitting datasets in the first part of evaluation.

#### Results based on the first workflow

Visual inspection of the raw spectra in Fig 1 suggests that there are a set of peaks in the mass range of *m/z* 1650–1850 that could act as a biomarker for identifying MRSA vs MSSA strains. To systematically obtain biomarkers, in the following, we will use careful pre-processing of this data and apply statistical learning methods.

**Fig 3:**
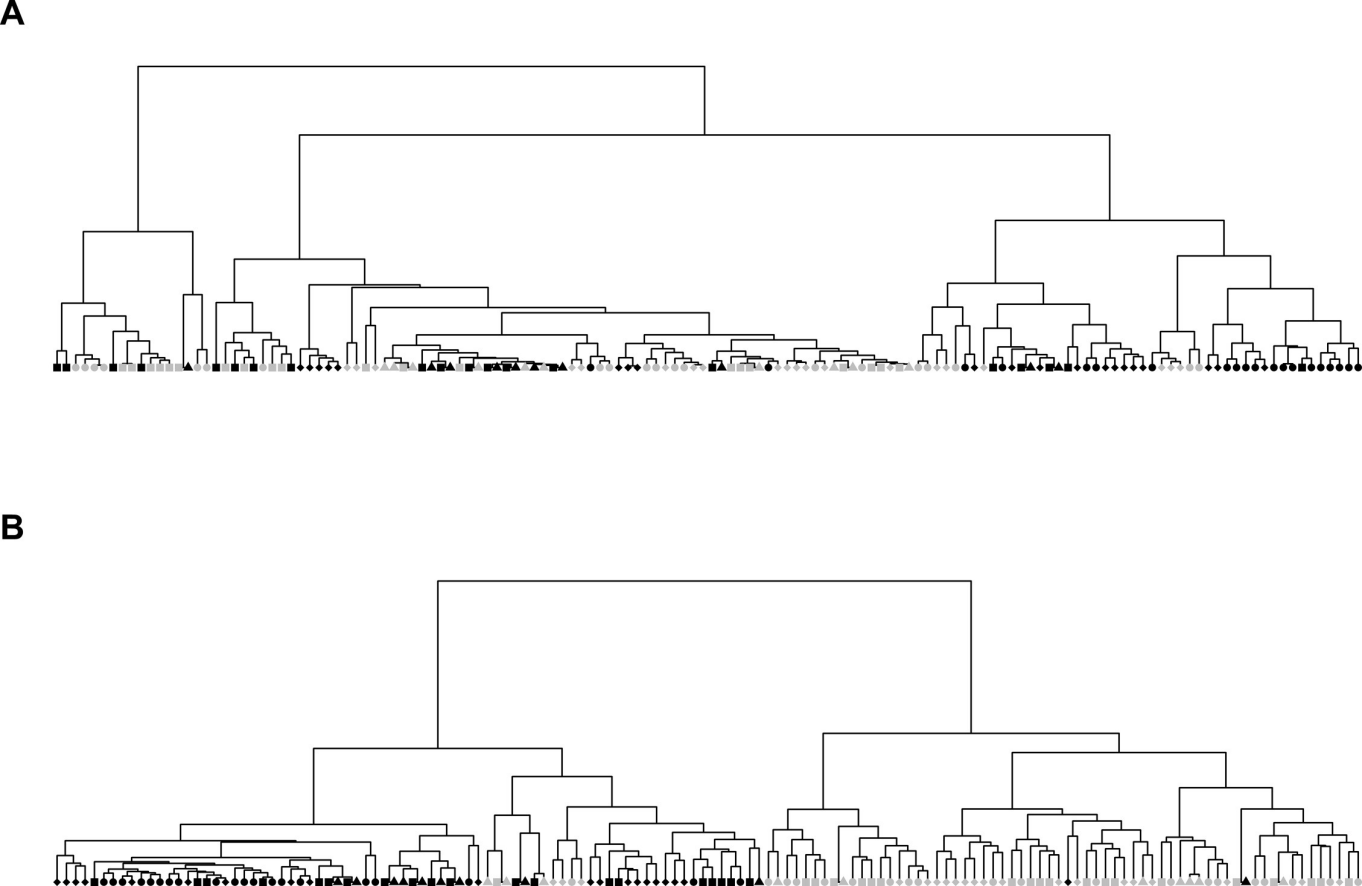
Results based on the first workflow. Dark circles: MSSA samples, grey circles: MRSA samples. Nodes with the same shape stand for the technical replicates. (A): Based on feature matrix extracted from MALDIquant, using correlation based distance metric in HC plot. (B): optimized binary matrix output from BinDA (using only top 100 features).

The first workflow (Fig 2A) is used to illustrate the presence of signal in the MS data for the identification of the two strains. Here, analysis is performed using the combined 7 datasets. After implementing the first workflow, the feature matrix is of dimension 140×2141. Then we applied Hierarchical Clustering Analysis (HCA). Based on this full set of features, MRSA and MSSA samples cannot be well separated, which is shown in Fig 3A. To investigate whether we can improve upon this result, we followed the analysis procedure [8] and proceeded to constructing an optimal binary feature matrix using BinDA. BinDA utilizes true labels of the data to find optimal threshold for each feature. The element in the feature matrix is changed to 1 if it is above the corresponding threshold and 0 otherwise. In addition, BinDA can also rank the features by comparing each group mean with the overall pooled mean. We used “binda.ranking” from the BinDA package for ranking features and applied HCA on the binary feature matrix using only the 100 top ranked features (hclust() with method = “ward.D2” and dist() = “binary”). The classification significantly improved, as shown in Fig 3B. These results suggest that there is a strong signal in the data which allows us to classify MRSA and MSSA samples [8]. Note that 69% of the top 100 ranked features obtained based on BinDA have *m/z* values above 1200, which further suggests that informative peaks mainly lie in the range above 1200. These findings are also consistent with what we observed in Fig 1, where peaks with *m/z* values less than 1200 tend to be noisier.

The first workflow used thus far requires all the datasets to determine the feature sets. In practice, we would like to determine the biomarkers based on some benchmarking datasets and then test it on new datasets. In the following, we will use our proposed second workflow to test reliability of this data in classification of MSSA and MRSA samples. Ideally, the top ranked features should not vary too much when training datasets change.

### Results based on the second workflow

#### The impact of choice of number of features on supervised learning performance

**Fig 4:**
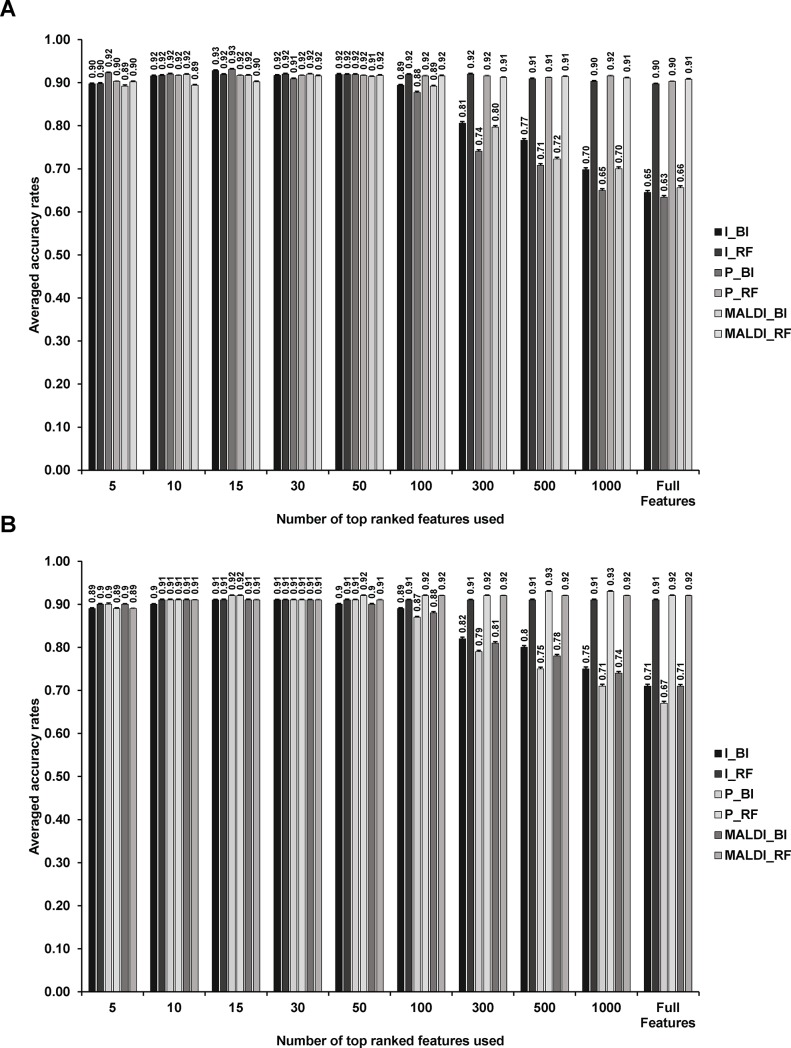
The impact of the top ranked features used on statistical model performance. Training and testing datasets were created by splitting either datasets (A) or samples (B). Error bars were calculated by either mean±sd/√32 (A) or mean±sd/√80 (B).

Firstly, we evaluated the effect of the top ranked features used on supervised learning. As Fig 4 suggests, BinDA seem to be not robust to the change in the number of features used in supervised learning. This may be due to the fact that in order to use BinDA for learning and testing, we have to transform the feature matrix extracted from the second workflow into a binary matrix as required by BinDA. The random forest method does not have this additional step. Interestingly, regardless of which way for creating training and testing datasets, the accuracy results are consistently higher than 90%. The highest accuracy rate we could achieve is 93% using “P_RF” method.

**Fig 5:**
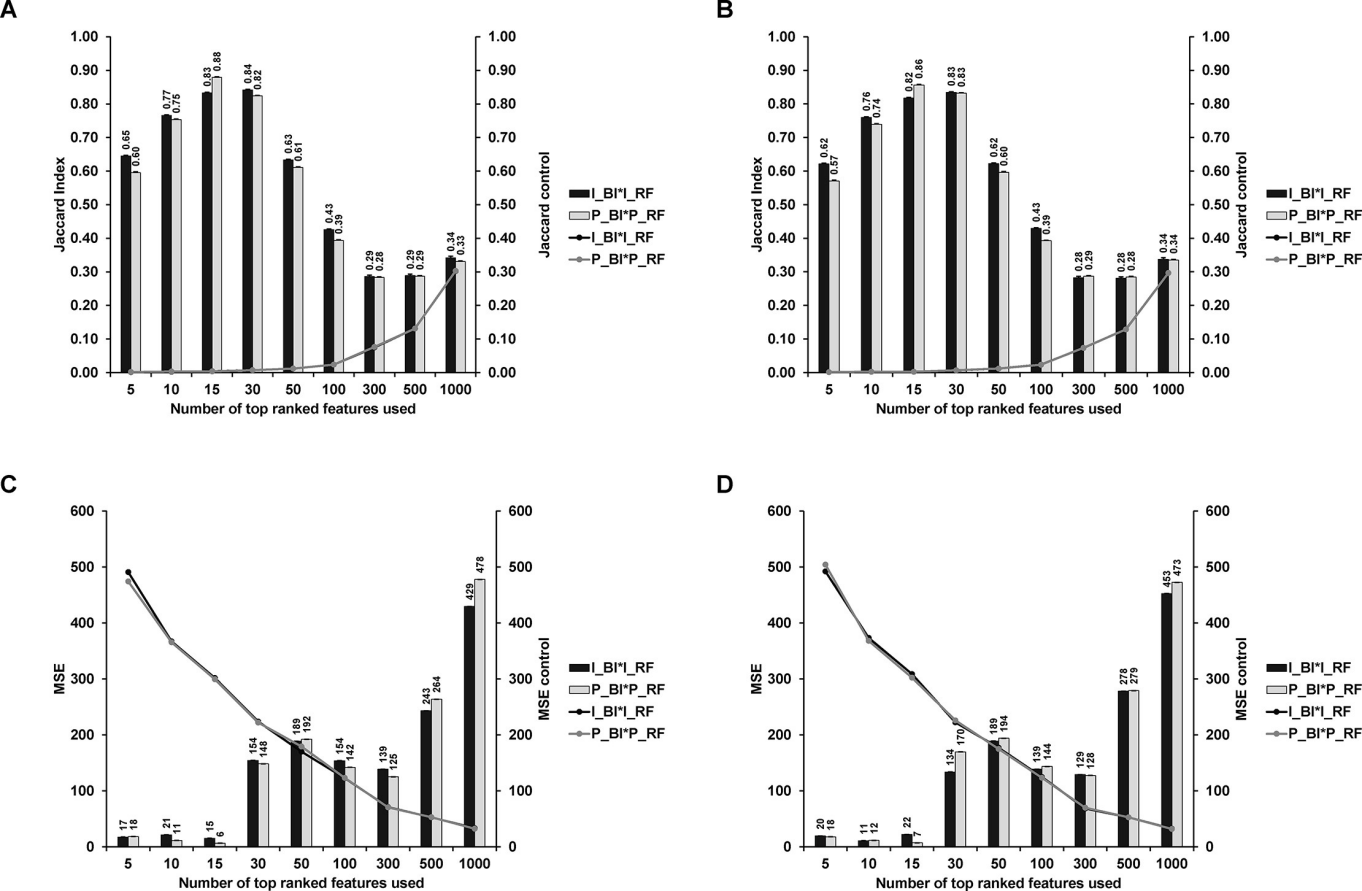
Comparisons of top ranked features using both Jaccard Index (A-B) and MSE (C-D). The error bars for the points on the solid lines (control) were obtained by mean±sd/√1000, but they are two small to be observed. Also note that for A and B, the line plots for the two workflows are very close to each other. Training and testing datasets were created by splitting either biological replicates (A, C) or samples (B, D). Top ranked features were compared between I_BI and I_RF (I_BI*I_RF) or P_BI and P_RF (P_BI*P_RF). Solid bars were calculated by either mean±sd/√32 (A, C) or mean±sd/√80 (B, D).

In order to check that whether the different performance results from variants of features, we compared features selected by either BinDA or random forest using both Jaccard Index (Fig 5A and 5B) and Mean Squared Error (MSE. Features reported from BinDA and random forest were ordered separately before computing MSE, Fig 5C and 5D). Jaccard index ranges between 0 and 1. The higher the Jaccard Index, the more similar the two sets are. For MSE, the lower the value, the more similar the two sets are. As control, the features of the same size were randomly selected from the full features. This bootstrapping was done 1000 times for each size of top ranked features. As the Fig 5 shows, when the size of top ranked features increases, the discrepancy between top ranked features extracted from BinDA and random forest increases regardless of intensity or peak area based feature matrix. The points on the solid lines represent controls obtained from bootstrapping. Note that most Jaccard Indices and MSE calculated from the top ranked features were significantly deviated from the controls. This observation is consistent with the results shown in the Fig 4, where the averaged accuracy rates obtained using BinDA or random forest are not consistent when the size of features is above 300. In short, the top ranked features reported by BinDA and random forest diverge when the number of top ranked features increases. Given this result, together with the suggestion that just a few features may be enough for achieving good classification using MS data[8, 20], we chose to focus on the top 100 ranked features in the following analysis.

#### Supervised learning using the top 100 ranked features

**Fig 6:**
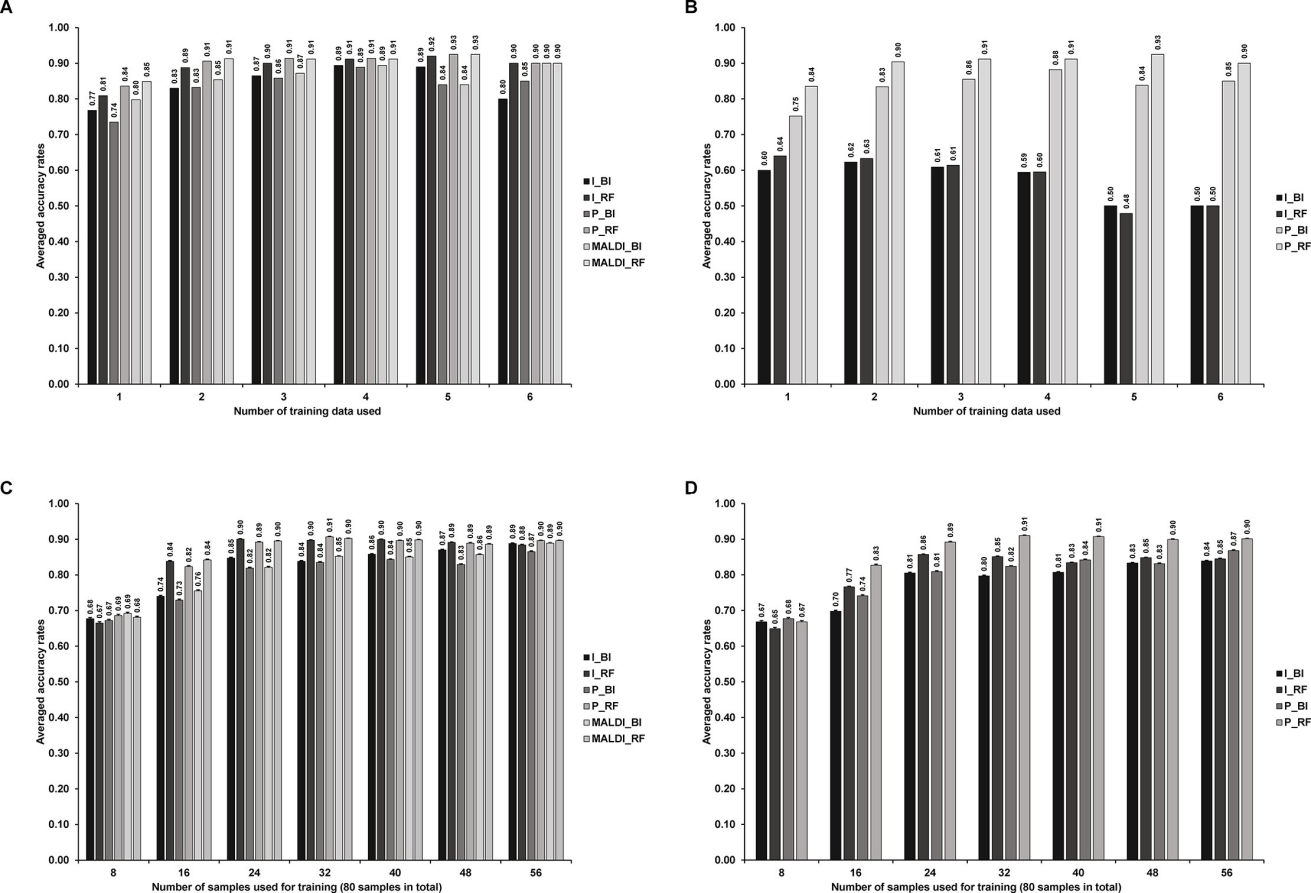
Classification accuracy rates as a function of the number of datasets (A-B) or samples (C-D) used in training procedure: (A): One of seven datasets was tested, while a subset of the rest datasets were for training. We also made sure that the technical replicate of testing data was not included in training. (B): Similar in (A), but we do not align testing data according to the reference peaks extracted from training data. (C) 24 out of 80 samples were used for testing, where the 80 samples come from the 4 biological replicates. The way we split samples made sure that the biological replicates of the same sample did not appear in both training and testing data. (D) Procedure similar to the panel (A), but here we do not align testing data according to the reference peaks extracted from training data. Error bars in panels (C-D) were calculated by mean±sd/√40. Error bars were not shown in panels (A-B) because different number of testings were performed in each case.

The averaged accuracy results as a function of the number of datasets or samples used in training procedure are shown in the Fig 6A and 6B (splitting by datasets) and Fig 6C and 6D (splitting by samples). The accuracy rate improves as we include more datasets or samples in training, which is as expected. Note that this is not the case when we used 6 datasets for training (Fig 6A and 6B), because we only have one choice for selecting 6 datasets for training while none of them are technical replicates of testing data. This analysis can help to determine how many datasets (biological replicates or samples) are required to reach maximal accuracy.

We next test the importance of alignment of new data according to the training data in the reference data-based workflow (Fig 2B. MALDI_* methods were not applicable here because both training and testing samples were pre-processed together). In Fig 6B and 6D, we apply the same procedure as we did in Fig 6A and 6C, but do not align testing data or new MS data according to the training data. For intensity based methods, accuracy rates decrease dramatically when training data obtained by splitting biological replicates (Fig 6B). In contrast using training data obtained using splitting samples, the accuracy rates decreased by just around 5% (Fig 6D). Overall, we find peak area based methods are more robust than intensity based methods.

#### Characteristic of the top ranked peaks

**Fig 7:**
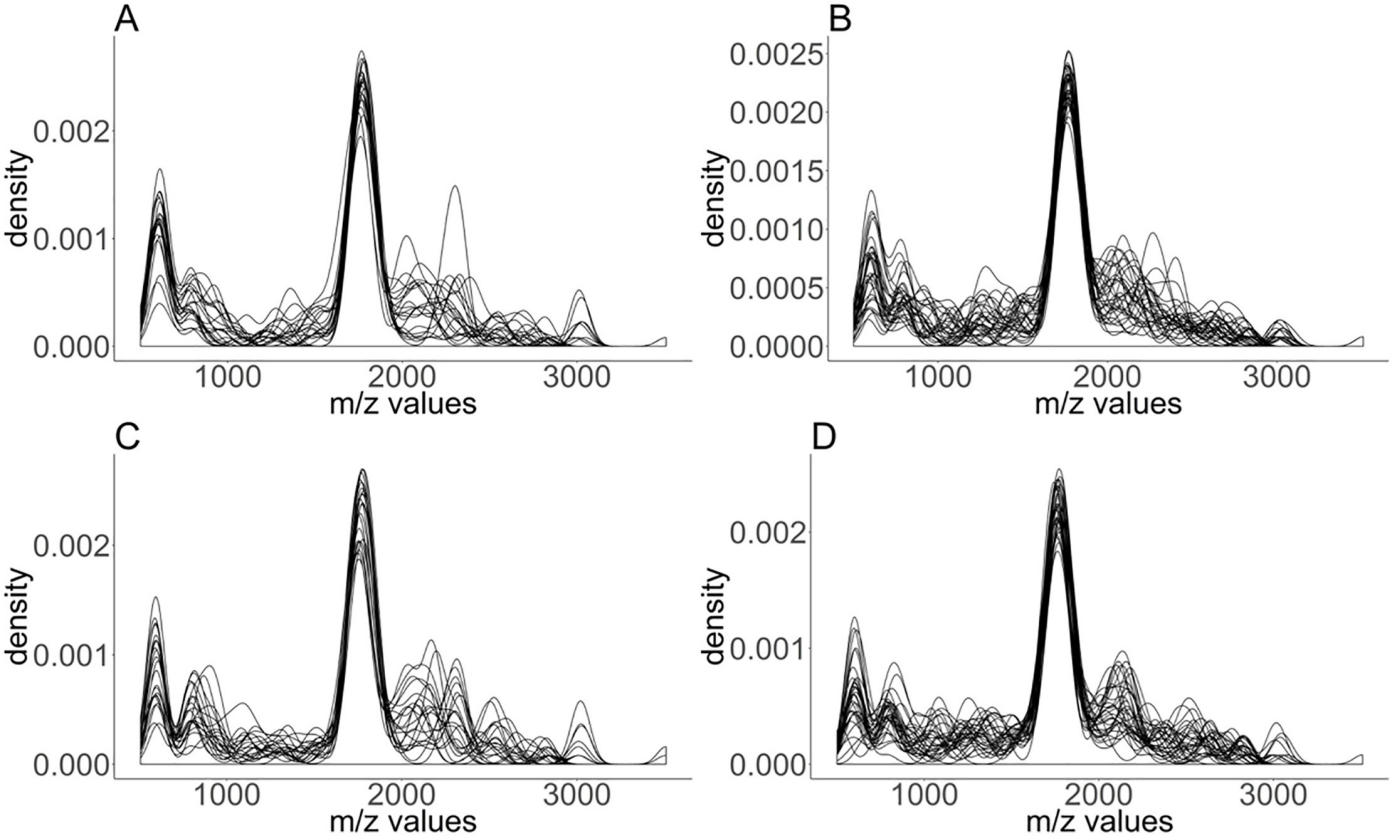
Density plot (bandwidth = 50) of the top 100 ranked features based on 4 variations of the second workflow. Here we pick 4 biological replicates from 7 datasets and repeatedly build model and rank features based on 3 biological replicates (A): I_BI, (B): I_RF, (C): P_BI, (D): P_RF.

The top 100 ranked features were extracted based on the Fig 4A (splitting datasets). Fig 7 indicates that most top-ranked features lie around 1600*m/z*. The result also indicates some signal in around 500*m/z*, but the density plot shows that the signal there is noisier. This is again consistent with what we observed in Fig 1. The top 100 ranked features can be found in the S1 Table.

## Discussion and conclusion

The use of MALDI-TOF MS to differentiate MRSA from MSSA has been already investigated. As for example, Edwards-Jones et al. used seven MSSA reference strains and seven MRSA clinical isolates for a *m/z* ranging from 500–10,000 using the matrix 5-chloro-2-mercaptobenzothiazole dissolved in water:methanol:acetonitrile 1:1:1 and containing 0.1% formic acid and 0.01 M 18-Crown-6 ether[21]. Du et al. identified 74% of 76 *S*. *aureus* strains and MRSA was correctly distinguished from MSSA using cluster analysis with a dendrogram[5]. Jackson et al. found that MSSA and MRSA in *S*. *aureus* had different peak intensities at *m/z* 3048, *m/z* 3086 and *m/z* 3124[22]. Drake et al. also proposed discrimination based on peaks observed at *m/z* 2302 and *m/z* 3871[23]. In this study, we implement a new route to diagnose and discriminate MSSA from MRSA by a combination of intact-cell MALDI profiling followed by careful pre-processing and supervised learning methods. By using 2,5-dihydroxybenzoic acid as matrix solubilized in organic solvents, we could identify strong peptide signatures from intact MSSA and MRSA.

In terms of preprocessing, in this study, we propose a modified workflow that allows defining a set of fixed reference peaks based on some predefined set of benchmarking dataset. Then, we describe how to map new testing datasets to the reference dataset. This approach is required for practical applications as one would like to classify newly obtained clinical samples based on a validated prior classification model. In the literature, although some methods such as “Peak Probability Contrasts” (PPC) [13] and BinDA [8] are both reference-based workflows, the detailed procedure of how to generate consistent feature matrices and map new datasets to these is not well described.

For the first workflow, if we want to apply supervised learning, we can pre-process all samples and then divide the final feature matrix to training and testing data separately. However, if we incorporate new data and apply the first workflow again, the features could change. The second workflow allows us to generate a consistent feature matrix based on a fixed reference and hence evaluate the robustness of the extracted features.

We evaluated the second workflow in three different ways. We have tested training data with or without biological replicates of testing data. In addition, we carefully avoid the potential impact between technical replicates in supervised learning by excluding technical replicates of testing data from training procedure. The promising results indicate that we are able to extract robust information from direct peptide fingerprints based MS data. We also learned it is optimal to use between 20–100 top ranked features and to use peak area based methods in combination with random forest for classification.

There still remains room for improving the workflow. As illustrated before, for reference based workflow, it is important to align new spectra according to the reference well. Only then can we further summarize MS data into the feature matrix. Hence a robust alignment method is necessary. Given that the pre-processing of MS data contains several steps and for each step there exists different methods, the workflow has potential to be further optimized.

As proof-of-principle, our integrative analysis offers a unique and unbiased data analysis and classification of new MALDI samples based on existing benchmarked datasets and could discriminate MSSA from MRSA with over 90% accuracy. Last but not least, our improved analysis workflow is generally applicable to identification by MALDI MS for any biological sample including bacteria, viruses and fungi. In summary, our approach promises a rapid and reproducible method to determine the resistance to methicillin in *S*. *aureus*.

## Supporting information

S1 Table List of Top 100 ranked features across the dataset. Tab 1, 2, 3 and 4 represent the Top 100 features for I_BI, I_RF, P_BI, P_RF conditions respectively.
(XLSX)

## Abbreviations

MALDI MS: matrix-assisted laser desorption ionization mass spectrometry
MSSA: methicillin-susceptible Staphylococcus aureus
MRSA: methicillin-resistant (MRSA) *Staphylococcus aureus*
